# Prone split-leg endoscopic-guided percutaneous nephrolithotomy: the surgeons perspective with A Gopro® view

**DOI:** 10.1590/S1677-5538.IBJU.2020.0524

**Published:** 2020-12-20

**Authors:** Yuri Botelho, Giovanni Scala Marchini, Manoj Monga, Fabio C. Torricelli, Alexandre Danilovic, Fabio C. Vicentini, Carlos A. Batagello, Miguel Srougi, William C. Nahas, Eduardo Mazzucchi

**Affiliations:** 1 Universidade de São Paulo Faculdade de Medicina Hospital das Clínicas São PauloSP Brasil Seção de Endourologia, Divisão de Urologia, Hospital das Clínicas, Faculdade de Medicina da Universidade de São Paulo, SP, Brasil.; 2 University of California DiegoCalifornia USA University of California, San Diego, California, USA.

## Abstract

**Introduction:**

To demonstrate the entire surgeon's point of view of a prone split-leg (PSL) endoscopic guided percutaneous nephrolithotomy (ePCNL) recorded with a GoPro® camera for standardization of the essential technical steps towards a successful procedure (1).

**Materials and methods:**

A 40y.o female patient presented with right flank pain for three years. She had previously been submitted to shock wave lithotripsy without success. Non-contrast computed tomography (NCCT) revealed a 2cm stone in the renal pelvis with 1400HU and stone-to-skin distance of 11cm (Guy's Stone Score 1). PCNL approach was chosen for providing higher chances of stone-free after a single procedure. Informed consent was obtained. The PSL ePCNL was uneventful with a single access in a mid-pole. The surgeon had a Full HD GoPro Hero 4® camera mounted on his head, controlled by the surgical staff with a remote control. All essential surgical steps were recorded.

**Results:**

Operative time was 90 minutes. Hemoglobin drop was 0.7g/dL. The post-operative NCCT scan was stone-free. The patient was discharged 24h after surgery. Kidney stent was left with a string and removed after 5days. The camera worked properly and didn't cause any kind of discomfort to the surgeon. The quality of the recorded movie was excellent.

**Conclusion:**

By recording the surgeon's perspective of an endoscopic urological procedure, we were able to provide a comprehensive understanding of the surgical technique by assembling the endoscopic, fluoroscopic, and operative field views. The GoPro® camera proved to be an interesting tool to document surgical procedures without compromising outcomes and has great potential for educational purposes.
